# DOSE – Global data set of reported sub-national economic output

**DOI:** 10.1038/s41597-023-02323-8

**Published:** 2023-07-03

**Authors:** Leonie Wenz, Robert Devon Carr, Noah Kögel, Maximilian Kotz, Matthias Kalkuhl

**Affiliations:** 1 Potsdam-Institute for Climate Impact Research, RD4: Complexity Science, Potsdam, DE 14473 Germany; 2 Mercator Research Institute on Global Commons and Climate Change (MCC), Berlin, DE 10829 Germany; 3 University of Potsdam, Faculty of Economics and Social Sciences, Potsdam, DE 14482 Germany

**Keywords:** Economics, Geography, Environmental health

## Abstract

Many phenomena of high relevance for economic development such as human capital, geography and climate vary considerably within countries as well as between them. Yet, global data sets of economic output are typically available at the national level only, thereby limiting the accuracy and precision of insights gained through empirical analyses. Recent work has used interpolation and downscaling to yield estimates of sub-national economic output at a global scale, but respective data sets based on official, reported values only are lacking. We here present DOSE — the MCC-PIK Database Of Sub-national Economic Output. DOSE contains harmonised data on reported economic output from 1,661 sub-national regions across 83 countries from 1960 to 2020. To avoid interpolation, values are assembled from numerous statistical agencies, yearbooks and the literature and harmonised for both aggregate and sectoral output. Moreover, we provide temporally- and spatially-consistent data for regional boundaries, enabling matching with geo-spatial data such as climate observations. DOSE provides the opportunity for detailed analyses of economic development at the subnational level, consistent with reported values.

## Background & Summary

Understanding what drives or impedes economic progress is of key importance to various aspects of global welfare such as the alleviation of poverty and reduction of inequality. Previous research has highlighted e.g. the role of institutions^1–3^, human capital^4,5^, geography^6–9^ and climate^10–14^. These factors can differ substantially not only across but also within countries. Yet, development indicators such as Gross Domestic Product (GDP) are typically available at the national level only (or provided at higher spatial detail for individual countries only), therefore limiting global analyses to the level of entire countries and hindering the detail and potential accuracy of their insights^15^. As such, there is a clear need for more spatially-detailed economic data on a global scale.

A few attempts to generate such data have already been made. One of the earliest versions of a global, sub-national economic data set comes from Nordhaus^6^, who developed a 1° × 1°-grid of per capita economic output to better understand the relationship between geography and economic activity. While innovative, this data set relies heavily on interpolation using population census data. In 2013, Gennaioli *et al*.^4^ published the first consistent sub-national economic data with administrative regions (as opposed to a grid), but at five year increments and partly also relying on interpolation across time. Building on the work of Gennaioli *et al*., Kummu *et al*.^16^ produced a “gap-filled” global gridded data set which also included the Human Development Index scores as well as GDP per capita and total GDP for all years between 1990 and 2015. Here, significant interpolation was necessary to fill gaps across time and space. Perhaps the closest example to the here presented DOSE data set^17^ in terms of spatial and temporal coverage is the Subnational Human Development Database by Smits and Permanyer^18^, which provides regional income per capita (amongst other indicators) for 1625 regions across 161 countries for the period 1990 to 2017. Unlike DOSE^17^, however, this data set relies on household surveys and interpolation between survey years for the majority of non-OECD countries. Other examples of sub-national GDP data include a gridded data set for China^19^ as well as another global grid (1 km × 1 km) constructed using nighttime light data as a proxy for GDP^20^. Although providing global coverage with consistent methodology, the use of nighttime light data as proxy for economic activity is subject to limitations. In particular, economic activities that do not necessarily emit additional nighttime light as they grow such as agriculture and forestry are not well captured if no additional data proxies are used^21^. This can lead to an underestimation of economic activity in rural, darker areas whilst overestimating it in areas with exceptionally high light^22,23^.

Sub-national economic data have been used in a variety of research related to regional development. For instance, Gennaioli *et al*.^24^ compared within- and across-country economic development, particularly focusing on the rate of income convergence. Cuaresma *et al*.^25^ assessed the determinants of economic growth in European regions. A third study, conducted by Lee *et al*.^26^, explored the connection between sub-national government institutions and economic development in 340 African regions, concluding a significant, positive relationship.

Despite these developments in the production and use of sub-national data, their suitability for global empirical analyses is still challenged by the use of interpolation across time and space. Whereas spatial interpolation is problematic for cross-sectional analyses, temporal interpolation is particularly of concern in the context of time series and panel regression analyses that exploit short-run variations in data, which would naturally be obscured by the use of interpolation spanning multiple years. In order to make accurate inferences about environmental and social phenomena that operate at shorter timescales, there is hence a need for non-interpolated data that better mirrors natural variation as it occurs.

To address these challenges, we present DOSE — the MCC-PIK Database Of Sub-National Economic output^17^. DOSE is a global data set in which aggregate and sectoral economic data from over 1,660 regions across the world have been harmonized without interpolation. Instead, DOSE^17^ builds on a unique collection of data from various data sources, including statistical agencies, yearbooks and academic literature - all of which have been recorded in Table 1. In total, the data span 83 countries with annual temporal coverage from 1960 to 2020 (although temporal coverage varies for different regions). This yields more than 46,000 observations, each containing economic output for a particular region in a given year. At minimum, each observation contains the total gross regional product (GRP). In the majority of observations, economic output data are also provided for three main sectors: agriculture, manufacturing, and services (referred to as sectoral GRP). In addition to regional economic output in local currency units (LCU) at current market prices as generally collected from the original data sources, we provide per capita estimates in LCU and US dollars at both, current and 2015 market prices to enable comparison across time and space. Importantly, unlike other similar data sets, DOSE^17^ does not use inter- or extrapolation across time to estimate values between or outside of the periods with real observations. Instead, it contains output values that reflect the figures reported by the raw data sources. Despite this, we are still able to provide data on an annual basis rather than incrementally, considerably improving the coverage compared to previous databases^4^. This annual resolution makes DOSE^17^ suitable not only for analysis of long-run economic development but also of short-run variations in output data. This is a distinct advantage that DOSE^17^ holds over previous iterations of global sub-national economic data sets, where short-term inter-temporal variation is often obscured. Furthermore, the economic data in DOSE^17^ reflect values in sub-national regions, avoiding the spatial interpolation used to produce gridded data sets^6,16^. The non-interpolated, sub-national detail in DOSE^17^ allows for a variety of potential research directions, with one prominent example being climate impact analyses. Weather and climate are rarely experienced homogeneously at the national level^27^. Consequently, the impacts of weather extremes on production may not be identified precisely by means of national-level data. DOSE^17^ can help better understand the heterogeneity in the resulting economic impacts. It is also suited for exploring more general political-economic questions of within-country development, or for case studies related to a specific economy or set of economic regions. Due to the large number of observations across both time and space, DOSE^17^ allows for robust cross-sectional and inter-temporal analyses (for example via fixed-effects panel regression).

**Table 1:** Broad structure of the raw data sources used.

Raw Data Source	Countries
EuroStat^35–37^	Belgium, France, Greece, Hungary, Italy, Lithuania, Norway, Poland, Portugal, Romania, Serbia, Slovenia, Spain, Turkey, United Kingdom
OECD Regional Statistics^38^	Brazil, Japan, Mexico, Sweden
National Statistical Institutes	Albania^39^, Argentina^40^, Australia^41^, Austria^42^, Azerbaijan^43^, Bahamas^44^, Belarus^45^, Bolivia^46^, Bosnia and Herzegovina^47^, Brazil^48^, Bulgaria^49^, Canada^50^, Chile^51^, China^52^, Colombia^53^, Croatia^54^, Czech Republic^55^, Denmark^56^, Ecuador^57^, Egypt^58^, Estonia^59^, Ethiopia^60–62^, Finland^63^, France^64^, Georgia^65^, Germany^66^, Greece^67^, Guatemala^68^, India^69^, Indonesia^70^, Iran^71^, Ireland^72^, Italy^73^, Japan^74^, Kazakhstan^75–77^, Kenya^78,79^, Kyrgzstan^80^, Latvia^81^, Malaysia^82^, Mexico, Mongolia^83^, Morocco^84^, Mozambique^85^, Nepal^86^, Netherlands^87^, New Zealand^88^, Nigeria^89^, North Macedonia^90^, Norway^91,92^, Pakistan^93,94^, Panama^95^, Paraguay^96^, Peru^97,98^, Philippines^99^, Poland^100^, Portugal^101^, Romania^102^, Russia^103^, Slovakia^104^, Slovenia^105^, South Africa^106–108^, South Korea^109^, Spain^110^, Sri Lanka^111^, Sweden^112^, Switzerland^113^, Tanzania^114^, Thailand^115^, Turkey^116^, United Arab Emirates, United Kingdom^117,118^, Ukraine^119^, Uruguay^120^, USA^121^, Uzbekistan^122^, Vietnam^123,124^
Literature	Australia^125^, Chile^126^, Colombia^127^, Hungary^128^, Indonesia^129^, Italy^130^, Laos^131,132^, Malaysia^133^, Mexico^134^, Pakistan^135^, Peru^127^, Philippines^136^, Russia^137^, Sweden^138^, Turkey^139^,Ukraine^140^, Uruguay^141^
UN Human Development Reports	Egypt^142–148^, El Salvador^149–154^, Honduras^155–160^, Uzbekistan^161–166^

For collecting the data, we made use of the two supra-national databases, EuroStat and the OECD Regional Statistics Database, as well as of records of numerous national statistical agencies. We also collected data provided in the literature. A detailed list of raw data per country can be found in Table S1 in the supplementary material. Countries appearing twice or more have various raw data sources.

**Fig. 1:**
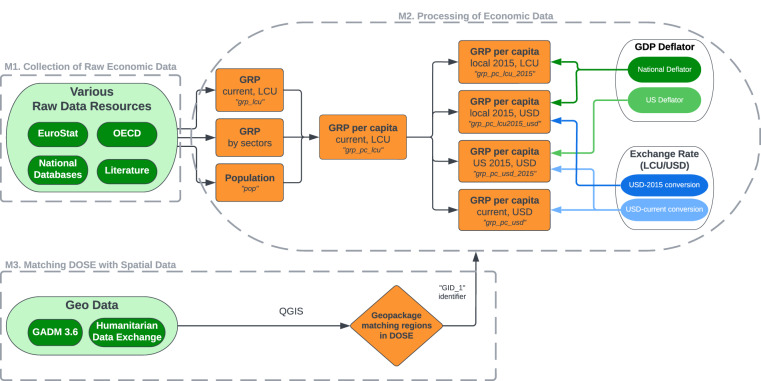
Schematic diagram of the generation of DOSE. DOSE was generated by collecting sub-national economic output and population data from various databases (M1). The data collection was then processed applying the following steps (M2): (1) harmonizing the aggregation of sectoral data, (2) generating per-capita values and (3) making them comparable between countries and over time using exchange rate and deflator data. Lastly, a corresponding geopackage was created for visualizing and matching the data to other sources (M3). Thus, DOSE provides a set of differently generated per-capita output variables, i.e. in local currency units (LCU) or US dollars (USD) as well as at current and constant 2015 prices (orange ellipse), such that users can select the variable that is most appropriate for their research question.

A schematic overview of the processes involved in the creation of DOSE^17^ is presented in Fig. 1 and a detailed description can be found in the Methods section. Annual, sub-national economic data were collected from various sources like EuroStat, the OECD database, national statistical institutes, and academic literature. Population data stem from the same data sources as the economic data, exchange rates from the FRED database and national price indices from the World Bank. The latter two were used to provide estimates that account for currency differences and inflation, respectively. We also provide geo-spatial data describing the administrative boundaries of the regions in DOSE^17^. These consider shifting administrative boundaries over time and can be used to flexibly combine DOSE^17^ with any other geo-referenced data set, for example to study the sub-national economic impacts of climate change. An earlier version of the DOSE data set has already been used in several of such impact-related studies, for example to assess the role of temperature and precipitation averages, variability and extremes in affecting economic growth^12–14,28^. Compared to this earlier version, we have here substantially updated and extended DOSE^17^, both in space and time as well as with respect to the inclusion of further variables reflecting different conversion and deflation methods.

In the following sections, we present a detailed account of the steps and methods involved in creating DOSE^17^, the data records it produced, a documentation of our technical validation, additional usage notes for those interested in its application, as well as access links.

## Methods

### Collection of raw economic data (M1)

The first step in the creation of DOSE^17^ consisted of the systematic collection of raw economic output data at the sub-national level. This step is represented schematically by the top left corner of Fig. 1, labeled M1. We used the first administrative division below national for consistency, corresponding to the *state*-level in the USA, *Bundesländer*-level in Germany, or equivalent. For countries in the European Union, the EuroStat database was used to obtain Gross Regional Product (GRP, total economic output) and Gross Value Added (GVA) by industry (sector-level output) data for all NUTS2 level regions. For OECD and some non-OECD countries, GRP data stem from the OECD Regional Database for TL2-regions. For the remaining countries, we used their respective national statistical databases, annual reports, or reviewed the literature to collect GRP and GVA by industry data. For some countries, GRP data were not available. Instead, we used the combined GVA of all industries as an estimate of GRP. A few other countries provided GRP by industry instead of GVA. If both total GRP and GVA by industry were not available, we used (household) income data as a proxy. This was the case for Ireland, Kenya, Pakistan, and Paraguay. For these countries, additional data such as average household size was necessary to calculate an estimate for total GRP.

In most of the raw data, sector-level GVA data (or GRP if GVA was not available) were pre-classified into economic activities based on the International Standard Industrial Classification of All Economic Activities (ISIC). Within DOSE^17^, these activities were further aggregated into three main sectors: agriculture, manufacturing, and services, as detailed in Table 2. Total GRP was generally collected separately, constituting the main estimate of aggregate GRP in DOSE^17^. Minor discrepancies between this total GRP value and the sum of the three sectoral outputs exist due to inconsistencies in the original national accounting. When estimating sectoral shares users are therefore advised to use the sum of output from the three sectors rather than the direct estimate of total GRP. In some cases, total GVA was used rather than total GRP due to a lack of data availability.

**Table 2:** Mapping of economic sectors.

Selection	Divisions	Description	Aggregation in DOSE
A	01–03	Agriculture, forestry and fishing	Agriculture
B	05–09	Mining and quarrying	Manufacturing
C	10–33	Manufacturing	Manufacturing
D	35	Electricity, gas, steam and air conditioning supply	Manufacturing
E	36–39	Water supply; sewerage, waste management and remediation activities	Manufacturing
F	41–43	Construction	Manufacturing
G	45–47	Wholesale and retail trade; repair of motor vehicles and motorcycles	Services
H	49–53	Transportation and storage	Services
I	55–56	Accommodation and food service activities	Services
J	58–63	Information and communication	Services
K	64–66	Financial and insurance activities	Services
L	68	Real estate activities	Services
M	69–75	Professional, scientific and technical activities	Services
N	77–82	Administrative and support service activities	Services
O	84	Public administration and defence; compulsory social security	Services
P	85	Education	Services
Q	86–88	Human health and social work activities	Services
R	90–93	Arts, entertainment and recreation	Services
S	94–96	Other service activities	Services
T	97-98	Activities of households as employers; undifferentiated goods- and services-producing activities of households for own use	Services
U	99	Activities of extraterritorial organizations and bodies	Services

The first three columns list the individual categories of the International Standard Industrial Classification of All Economic Activities (ISIC)^167^. The last column shows how we aggregated them into the three main sectors for which we provide sectoral GRP data, agriculture, manufacturing/industry and services.

If the raw data source differed for a given country over time or the respective institute provided a different structure of GVA by industry, we ensured to aggregate over similar categories to have consistent sectoral coverage over time. With Ecuador, for example, the original raw data were split into economic activities that were more specific than those in the ISIC classification. Agricultural output in particular was separated into eight categories based on crop and animal type. In such cases, we made common-sense judgements to determine to which of the three sectors in DOSE^17^ these more specific activities should be allocated. For some countries, sectoral data were unavailable.

**Fig. 2:**
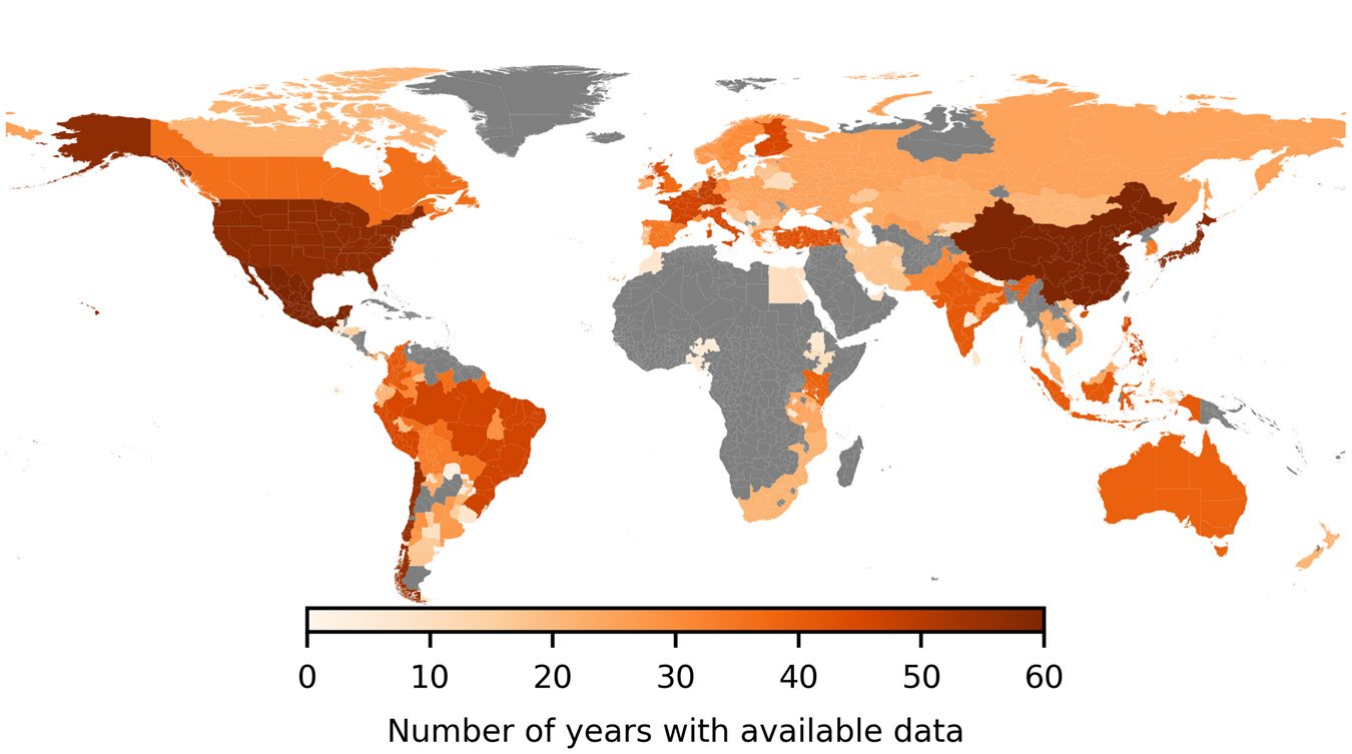
Regional and temporal coverage of aggregate GRP data in DOSE. The regional coverage within a country is in most of the cases similar. The longest time series are available for regions in China, USA and Mexico. Temporal data coverage varies across Latin-American regions and is sparsest for African regions. In Africa, GRP could generally only be assembled for a small number of countries.

**Fig. 3:**
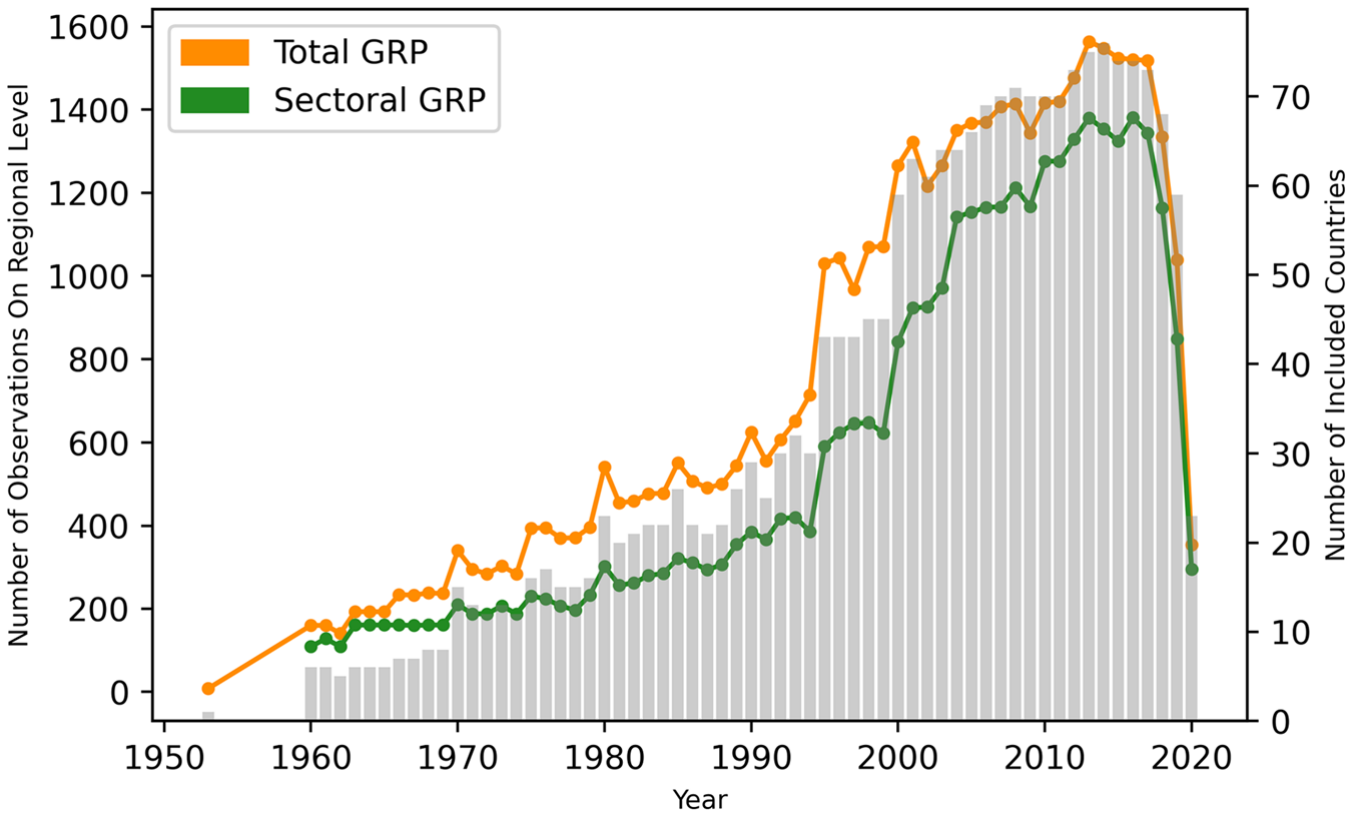
Coverage of GRP Data in DOSE over time. The plot shows the number of observations for total (orange) and sectoral (green) GRP per capita over time (left axis) and the number of countries (grey) for which those data are available in each year in DOSE (right axis).

In total, we were able to assemble economic output data for 1,661 regions in 83 countries around the globe. The full spatial coverage is represented on the world map in Fig. 2. Coverage of GRP data over time is shown in Fig. 3. As can be seen in Fig. 2, temporal coverage varies across regions. There are comparably long time series with more than 40 observations per region across Northern America, Europe, East Asia, Oceania, Latin-America and India. By contrast, data were only available from 1990 or later on-wards for Russia and countries of the former Soviet Union. Data coverage is particularly sparse for African and Middle-Eastern countries where official reports of sub-national economic data are largely lacking. Kenya constitutes an exception here as data on labor earnings (1969–2012) and GRP (2013–2019) are available from 1969 onwards. Furthermore, we were also able to collect sub-national economic data for more than 20 years for several South-east African countries (South Africa, Mozambique, and Tanzania). In comparison, data for North African countries were available for less than ten years per region. Table 1 provides an overview of all raw data sources and Table S1 in the supplementary material a detailed list of raw data per country. In the cases that raw data sources changed over time, we document these structural changes with a categorical variable “StructChange”, which e.g. takes the value of 1 in those years where a new raw data source has been introduced for the first time. Raw data were generally collected at current market prices in local currency.

### Processing of economic data (M2)

In order to compare economic data across regions, we calculated per capita figures, using sub-national population estimates from the same data sources as the economic output. Total and sectoral GRP data were divided by the regional population to obtain per capita output in current market prices. We include the population estimates in the final data set to facilitate conversion between per capita and absolute figures.

An overview of all auxiliary data used and variables constructed in the processing step can be found on the right side of Fig. 1, labeled M2. All variables contained in DOSE^17^ are listed in Table 3. In addition to nominal per-capita sectoral and gross regional products in local currency units, we also provide estimates in constant 2015 LCU values as well as in current and constant US dollars (along with the data used for these conversions), thus enabling the use of our data in a broad range of contexts and applications.

**Table 3:** DOSE variable descriptions.

Variable	Description
country	Full-length country name
GID_0	3-digits ISO-Code
region	Primary region name
GID_1	GADM-identifier for level-1 administrative unit, or customly created identifier as indicated in Table 4 (in the same format as GADM-identifier)
year	Calendar year
grp_lcu	Gross regional product in local currency and current prices
pop	Regional population estimate
grp_pc_lcu	Gross regional product per capita in local currency and current prices
grp_pc_usd	Gross regional product per capita in US dollar and current prices
grp_pc_lcu_2015	Gross regional product per capita in local currency and 2015 prices
grp_pc_usd_2015	Gross regional product per capita in US dollar and US 2015 prices (conversion method given in eq. (1))
grp_pc_lcu2015_usd	Gross regional product per capita in local 2015 prices and converted to US dollar using the 2015 exchange rate (conversion method given in eq. (2))
cpi_2015	Worldbank Consumer Price Index with base year 2015
deflator_2015	Worldbank national GDP deflator with base year 2015
fx	FRED market exchange rate (local currency to one USD)
ppp	Purchasing Power Parity exchange rate (local currency to one international dollar)
StructChange	Categorical variable indicating the start of a new data source (1), changes in administrative boundaries (2), and when regional time series were extended to a previous version of DOSE (3)
T_a	Area-weighted annual mean temperature
P_a	Area-weighted annual total precipitation

List of all variables provided within DOSE (first column) and accompanying description (second column). If available, GRP data are also provided for the different production sectors: agriculture, manufacturing/industry and services, indicated by the prefix *ag_*, *man_* or *serv_*.

**Fig. 4:**
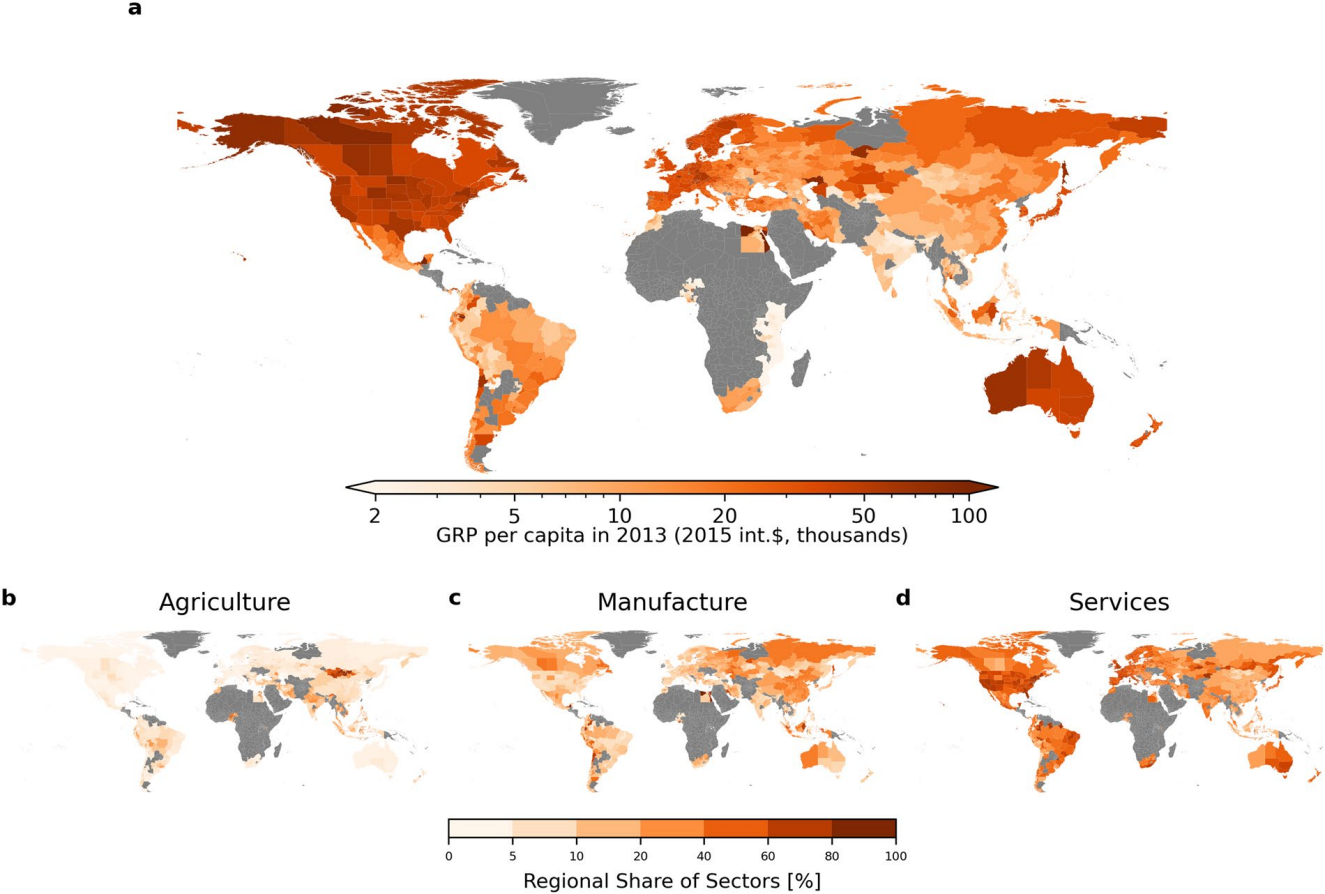
Distribution of GRP and sectoral GRP shares around the world. Panel a: PPP-adjusted GRP per capita in 2013 (2015-international dollars, thousands). PPP-adjusted output values are used here for this cross-region comparison. High incomes are concentrated in North America, Western Europe, Australia and Japan, but there are considerable within-country differences. Panels b to d: Sectoral shares of 2013-GRP (agriculture, manufacturing/industry and services). Agricultural shares are generally larger in low income regions, among them many in Asia, Africa, and South America. In several middle- and high-income countries, the services sectors are strongest in coastal regions.

**Fig. 5:**
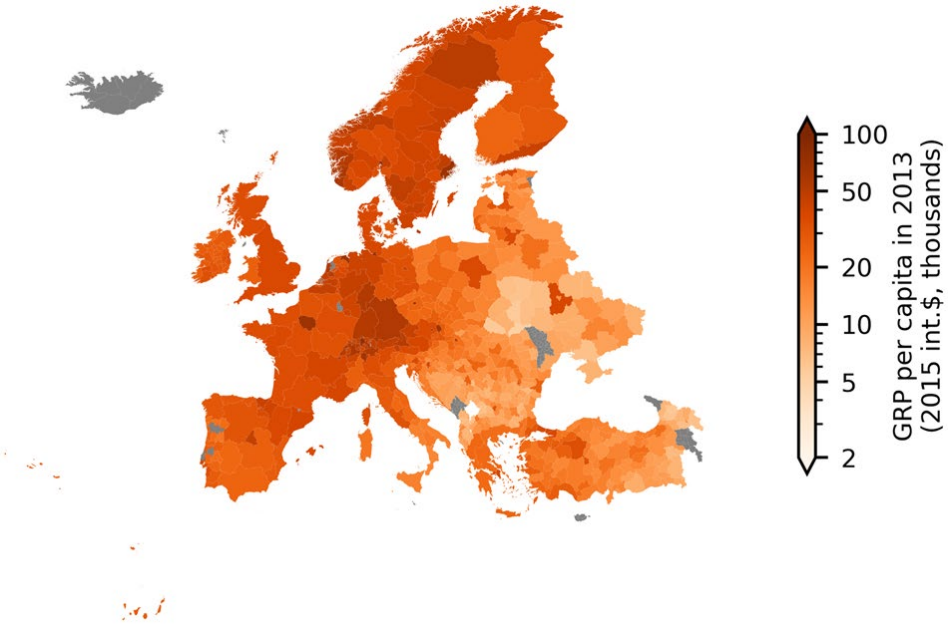
PPP-adjusted GRP per capita in 2013 (2015-international dollar, thousands) by European regions. PPP-adjusted output values are used here for this cross-region comparison. Capital cities generally boost regional GRP. For Spain and Italy, the southern regions tend to have lower incomes than their northern counterparts, whereas in Germany differences in income levels between western and eastern regions continue to reflect the former division of Germany. In eastern and south-eastern Europe where income levels are considerably lower compared to the rest of Europe, income levels are higher for those regions closer to western European countries.

Two types of data were used in these conversions, namely, market exchange rates and national-level GDP deflators. Both indices consistently account for historic currency reforms when necessary. Market exchange rate data were taken from the FRED database of the Federal Reserve Bank of St. Louis, specifically the “Penn World Table 10.0”^29^. The specific format of these data is national currency units per US dollar, collected annually. Using these annual exchange rates, we converted sectoral and total GRP in local currency per capita to US dollar equivalents. An alternative option is to use a Purchasing Power Parity (PPP) exchange rate as it tends to better reflect price differences in consumer goods across countries. Since non-tradeable goods and services are cheaper in lower-income countries, the use of market exchange rates can underestimate the purchasing level of incomes in these countries. Another limitation of using market exchange rates to convert to a common currency is that under political influence, they may not accurately reflect a nation’s real economy. For example, in countries that have pegged their currency to the dollar, the market exchange rate is subject to government regulations that could misrepresent true purchasing power. In such contexts, PPP exchange rates may offer a more accurate conversion. However, due to the interpolation of PPP between survey years which occur in approximately 6-year increments^30^, we opted for market exchange rates to avoid introducing an interpolation bias which would pose particular problems for time-series and panel analyses. In the context of cross-sectional analyses, by contrast, the use of PPP-based values may be favorable. For example, when visualising the distribution of incomes across regions in Figs. 4, 5, as well as when comparing our data to another database which is based on PPP values (Fig. 7), we show PPP-adjusted values, i.e. for the generation of these values PPP instead of market exchange rates were used. We hence also provide PPP exchange rates within the DOSE data set^17^ to enable PPP-based conversions.

In order to convert current prices into constant 2015 prices, we used national GDP deflator data from the World Bank. By using one global data source, we minimize the risk of country-specific measurement irregularities. As the World Bank deflator data set originally used different base years for each country, we harmonized the deflator time series to base year 2015. We chose to use a GDP deflator because unlike a consumer price index (CPI) it also takes into account price changes in purchases made by businesses, governments, and foreigners in addition to those made by regular consumers. Furthermore, a GDP deflator ignores price changes from importers. Unfortunately, deflator data are generally unavailable at the sub-national level, such that regional variations in prices are not accounted for.

In general, there are two approaches to make economic data comparable between regions and across time using market exchange rates and national deflator data, both of which we produced with our data. The approaches differ in the order with which they deal with the inter-national and inter-temporal components of currency comparison and each have their strengths and shortcomings. The first approach given in Eq. 1 converts current per-capita GRP of region *r* in year *y* in local currencies, *grp_pc_lcu*_*r, y*_, first into current US dollars, using the market exchange rate fx_r,y_ for year *y*, and then applies the US deflator with base year 2015, deflator_2015_USA,y_, to obtain values at constant prices.

1 $${\rm{grp}}\_{\rm{pc}}\_{\rm{usd}}\_{{\rm{2015}}}_{{\rm{r,y}}}=\mathop{\underbrace{{\rm{grp}}\_{\rm{pc}}\_{{\rm{lcu}}}_{{\rm{r,y}}}\times \frac{1}{{{\rm{fx}}}_{{\rm{r,y}}}}}}\limits_{{\rm{grp}}\_{\rm{pc}}\_{{\rm{usd}}}_{{\rm{r,y}}}}\times \frac{100}{{\rm{deflator}}\_{{\rm{2015}}}_{{\rm{USA,y}}}}.$$

With such an approach the lack of sub-national price indices is bypassed, but under the assumption that goods are perfectly tradeable between countries. Similar procedures for sub-national entities have already been applied in some contexts. For instance Kalkuhl & Wenz^12^ used GRP in current US dollars and applied region-specific polynomial time trends and year-fixed-effects to control for changes in regional prices.

A second approach, given in Eq. 2, controls first for local nominal price changes prior to applying the market exchange rates for the year 2015.

2 $${\rm{grp}}\_{\rm{pc}}\_{\rm{lcu2015}}\_{{\rm{usd}}}_{r,y}=\mathop{\underbrace{{\rm{grp}}\_{\rm{pc}}\_{{\rm{lcu}}}_{r,y}\times \frac{100}{{\rm{deflator}}\_{{\rm{2015}}}_{r,y}}}}\limits_{{\rm{grp}}\_{\rm{pc}}\_{\rm{lcu}}\_{{\rm{2015}}}_{{\rm{r,y}}}}\times \frac{1}{{{\rm{fx}}}_{r,2015}}.$$

In particular, the local national deflator, deflator_2015_r,y_, is used to calculate GRP in constant 2015 prices, after which the US dollar exchange rate in 2015, fx_r,2015_, is applied to convert to constant US dollars. The advantage of the second approach lies in its correction of national inflationary tendencies that are not well represented by market exchange rates. However, the application of national deflators as the main correction for inter-temporal price changes may not well reflect differential within-country price changes which emerge over time^31^. The use of sub-national price indices could mitigate this issue, but such data are not currently available for the majority of regions.

We here provide estimates resultant from both methods as well as nominal values in either LCU or US dollars such that users can employ the variable that is most suitable in their context. For the same reason, the DOSE data set^17^ also contains Worldbank’s CPI data with base year 2015. In the section on technical validation, we compare both conversion methods to a previous sub-national database^24^ which instead used sub-national GRP in constant local prices to downscale national GDP in constant, US dollars. We find that both methods compare similarly well, with slightly greater consistency when using our first method for accounting for currency and inflation changes.

**Fig. 6:**
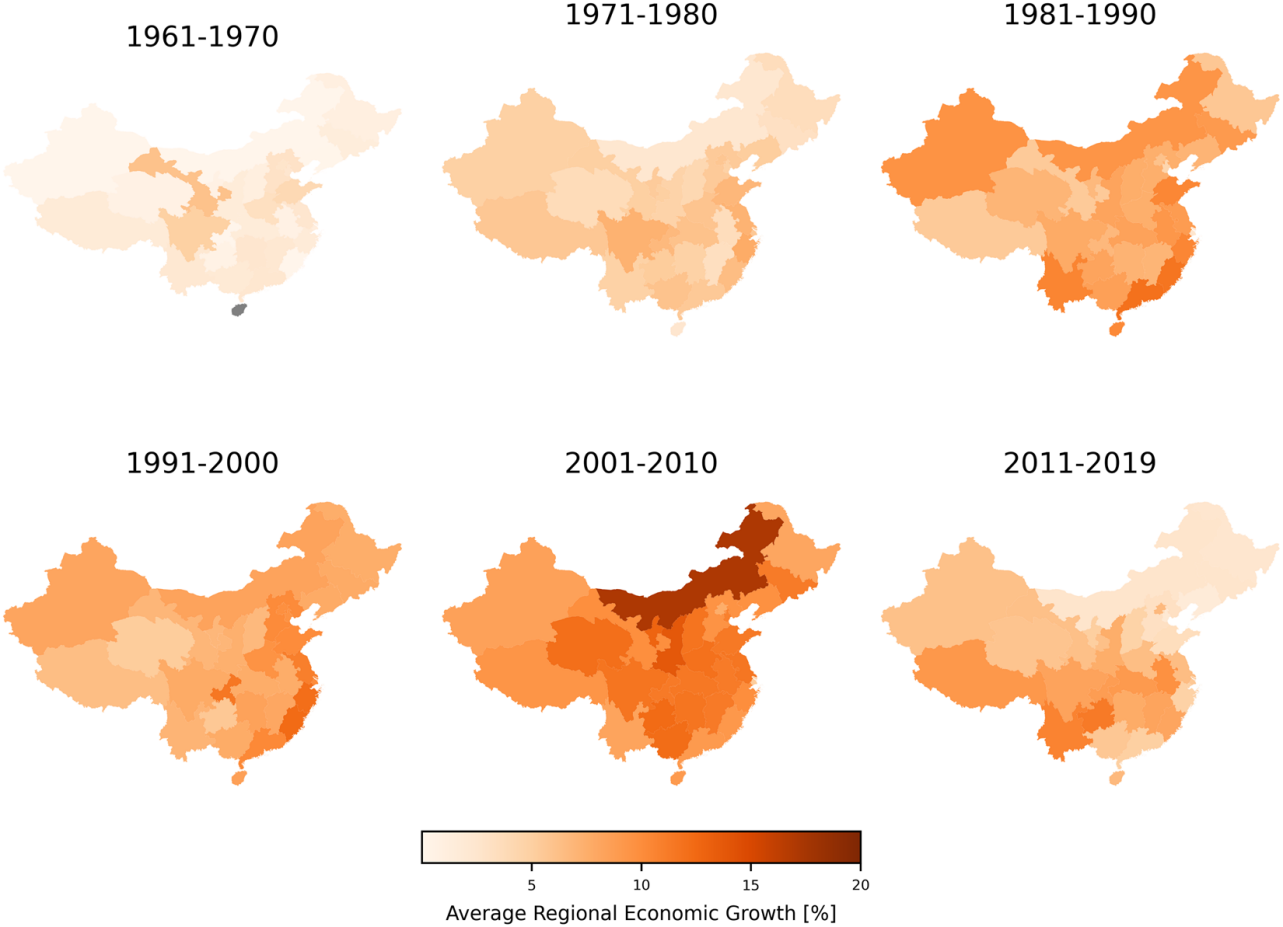
Average growth rates in constant USD for Chinese regions by decade (1960–2019). Economic growth sped up in the 1980s and peaked during the 2001–2010 period. Regional growth was strongest across south-eastern coastal regions through the 1980s and 1990s, but in the 2010s stronger development occurred inland. Over the last decade, economic growth considerably decreased especially for the north-eastern regions.

The resulting economic variables can be used for a variety of analyses. We provide a few basic examples here. Figure 4 shows how GDP and sectoral output were distributed across the world in 2013. In Fig. 5 one can observe more detailed within-country differences for the example of Europe. Figure 6 demonstrates the possibility to examine trends in economic growth over time, here exemplified for Chinese provinces.

### Matching DOSE with spatial data (M3)

To accompany the economic data in DOSE^17^, we provide unique identifiers for each region that can be used to match them to the relevant administrative boundaries (see section M3 in Fig. 1). Specifically, we provide two identifiers for each region: one for the first subnational level boundary, GID_1, and a second for the national level it belongs to, GID_0. Users can find spatial data at the same regional level as our economic data (first sub-national level) for all but eight countries by using the Database of Global Administrative Areas (GADM, https://gadm.org/download_country_v3.html), in particular version 3.6.

In the eight exceptional countries, the regions listed in a given country’s economic data did not match with any of the regions provided by GADM’s first sub-national level. This was due to one of two reasons: (i) the economic data were collected at a level that was either finer or coarser than any of the administrative levels covered by *GADM level 1*, or (ii) region names and administrative boundaries had changed either over time or between the time that the economic data and GADM version 3.6 were published. In such cases, we provide the spatial data ourselves. Shape-files were gathered from alternative sources, like the Humanitarian Data Exchange, or created manually (i.e., by aggregating smaller administrative boundaries). For example, in Macedonia, *GADM level 1* lists 85 sub-national regions whereas we collected economic data for only eight regions. Here we aggregated the smaller regions from *GADM level 1* to match the larger regions for which economic data were available (using QGIS and sources such as http://citypopulation.de/en/northmacedonia/cities/). Although this division does not reflect the official administrative division of the first sub-national level but rather the division of statistical regions, we preferred to provide a broader regional division instead of none. We then created GID_1 identifiers for these ‘custom’ regions (starting from MKD.100_1, MKD.101_1, etc. to ensure that there was no overlap with the official *GADM level 1* identifiers, which go from 1–85).

In rare cases, the set of sub-national regions in a country’s economic data changed over time. In the case of Morocco, for instance, the set of regions in the economic data for 2007–2013 differs from those for 2014–2019. To preserve this distinction, we treat the two sets of regions as separate entities, providing spatial data for the regions not found in GADM 3.6, and unique identifiers for both sets of regions. In Morocco’s case, *GADM level 1* data could be matched to the regions for 2014–2019, whilst the Humanitarian Data Exchange was used to acquire spatial data for the 2007–2013 regions, which were then given a unique GID_1 identifier.

Table 4 shows the full list of countries whose economic regions did not initially match with the *GADM level 1* division or any other level. Spatial data from *GADM level 1* can be merged with the spatial data from Table 4 regions to produce a comprehensive shapefile with geometry for each region in DOSE^17^. The extra procedures applied to the eight exceptional countries (e.g. Macedonia and Morocco) ensure spatial and temporal consistency with the observations in DOSE^17^.

**Table 4:** Sources of spatial data. For the countries listed above, the regional level of our economic data does not match with first level sub-national regions in spatial data from GADM version 3.6. In these cases, we provide spatial data taken from the sources listed above. For Bahamas and Macedonia, we merged *GADM level 1* regions manually in QGIS to match the economic regions.

Country	Explanation
Bahamas (two regions)	merged GADM-1 regions to match statistical regions
Kazakhstan	matching spatial data downloaded from HumData Exchange
Macedonia	merged GADM-1 regions to match statistical regions
Morocco (regions for 2013–2019)	matching spatial data downloaded from HumData Exchange
Nepal	matching spatial data downloaded from HumData Exchange
Philippines	matching spatial data downloaded from HumData Exchange
Portugal (five regions)	matching spatial data downloaded from EuroStat
Sri Lanka	matching spatial data downloaded from HumData Exchange

### Matching climatic data

Assessments of economic climate impacts are a typical example of using DOSE^17^ in conjunction with detailed geo-spatial data^12–14^. Within the data set, we provide estimates of sub-national annual mean temperature and annual total precipitation having aggregated high-resolution climate data from the ERA-5 reanalysis^32^ to the administrative boundaries described in the previous section (see Matching DOSE with Spatial Data). We calculate annual mean temperature and annual total precipitation from daily 2-m air temperatures and daily precipitation totals at the grid-cell level (0.25-by-0.25 degrees), before aggregating to the sub-national administrative boundaries using an area-weighted average. In case a grid cell is split between two regions or a region and the ocean, we assess the proportion of the grid-cell that falls within the administrative boundary of a region and use this to weight its contribution to the sub-national average of that region.

A sample visualization using these climate variables can be found in Figure S1 in the Supplementary Information.

## Data Records

The data set and corresponding spatial data are available on Zenodo at the following repository 10.5281/zenodo.7573249^17^. The main file *DOSE_V2.csv* contains the raw economic data, typically gross regional product at current market prices in local currencies, *grp_lcu*, and population data, *pop*, as collected from the different data sources for 1,661 sub-national regions in 83 countries from 1960–2020. For Australia, regional GRP data are also available and included for 1953. If there was a change in either the data source or the administrative boundaries of a region, this is indicated by the *StructChange* column. The main data file further comprises the processed economic data, i.e. per-capita values at current and constant prices in local currencies and USD, as described in the Methods section (*grp_pc_lcu*, *grp_pc_lcu_2015*, *grp_pc_lcu*2015*_usd*, *grp_pc_usd* and *grp_pc_usd_2015*). Deflator data, *deflator_2015*, and exchange rates, *fx*, used to generate these different economic variables are given as well. GRP data are also provided at the sector-level for the agriculture, manufacturing/industry and services sectors, indicated by the prefixes *ag_*, *man_* and *serv_*, respectively. To facilitate further analyses, 2015 Consumer Price Indices, *cpi_2015*, and Purchasing Power Parities, *PPP*, as well as annual temperature, *T_a*, and precipitation, *P_a*, data are included. For each country and region, there are unique identifiers, *GID_0* and *GID_1*, given. Each column of the main file *DOSE_V2.csv* is described in full in Table 3.

The second file, *DOSE_shapefiles.gpkg*, is a composite shapefile (packaged in the geopackage format) that contains administrative boundaries for the eight exceptional countries listed in Table 4 for which spatial data cannot be obtained from GADM version3.6, along with the region names and unique identifiers that can be used to match them with *DOSE_V2.csv*.

## Technical Validation

The technical validation of DOSE^17^ was carried out in two distinct processes: (1) manual validation and (2) comparison with similar, pre-existing data. Each of these steps is explained in detail in the subsequent sections, followed by a brief discussion of the data set’s limitations.

### Manual validation

To ensure data quality, we manually inspected the timeseries throughout the data collection process. This involved checking for missing values, outliers, or unexpected patterns in the data, as well as identifying any discrepancies or errors that needed to be corrected. When collecting economic and population data at the sub-national level, we ensured the sum of all regions resembled the indicator’s value at the national level. We also examined possible differences between the sum of the computed sectoral GRP values and their total GRP value in a given year, which may occur because of different considerations of financial intermediaries or product taxes by the statistical institute. We determined that there were no instances where this gap was unreasonably large, but researchers are advised to compute sectoral shares based on the sum of the three sectors instead of using total GRP as base value. We validated the time series’ consistency by closely inspecting any jumps in the data. If one occurred, we compared it with data from other regions in the same country. If it appeared to be a year of economic slowdown for the whole country, we compared our data with national GDP records provided by other sources, such as World Bank, to exclude any error in the raw data. For example, Egyptian regions showed a strong decrease in economic production between 2000 and 2003 which we initially flagged as questionable, but could then confirm by comparing it to the national GDP values reported by the World Bank. Irregularities also occurred when the raw data were available in PDF format only (e. g. Kenya, Vietnam). For very large data sets, we made use of software that converted these data to spreadsheet format. In these cases, validating the conversion’s accuracy retrospectively was of high relevance. A common mistake of the software was adding an additional 0 which increased the data by one decimal power. Comparing the indicators to those at the national level revealed these cases reliably.

After having collected and merged the sub-national data, we used the coefficient of variation (CV), i.e. the ratio of the standard deviation to the mean, in output data for each region over time as a simple metric to further identify potential outliers (see Figure S2 in the Supplementary Information). In most cases, coefficients of variation are far below 1, with a few exceptions. Particularly large values were observed in China (*CV*∈[1, 1.5]), but these were found to reflect the exceptionally long time periods of large economic growth rates since the 1960s rather than inconsistencies in the raw data. Similarly large values for Kenya were also identified (*CV*∈1,2), and in this case found to arise due to the change in data sources from labor earnings data to GRP between 1999 and 2013, changes which are accounted for by our use of categorical variables indicating structural changes (see Methods section “M1. Raw Economic Data Collection”).

### Comparison to Pre-existing data sources

As outlined in the background section, some data sets of sub-national economic output are already available, offering the opportunity to compare these data to DOSE^17^ for a general validation of our estimates. The most comparable source is that provided by Gennaioli *et al*.^24^, which constitutes values for sub-national entities (rather than gridded values) but only at five year increments, for some regions beginning in 1950. However, Gennaioli *et al*.^24^ (referred to as G2014 from hereon) used a different approach to account for inflation and currency exchanges. They first estimated the share which each region contributes to the national sum of regional GRP in current, local prices. This share was then used to downscale national GDP in constant, PPP international dollars to the sub-national level at five year increments. Since we have used different approaches to address inflation and currencies as discussed in the Methods section, neither data set should be considered the “ground-truth”, but the comparison should nevertheless facilitate confidence in the general level of consistency. To make our data as comparable as possible to G2014, we use PPP-exchange rates rather than market ones. We compared the indicator of G2014 to both of our PPP-adjusted variables (one for each conversion technique outlined in the section “Processing of Economic Data”).

**Fig. 7:**
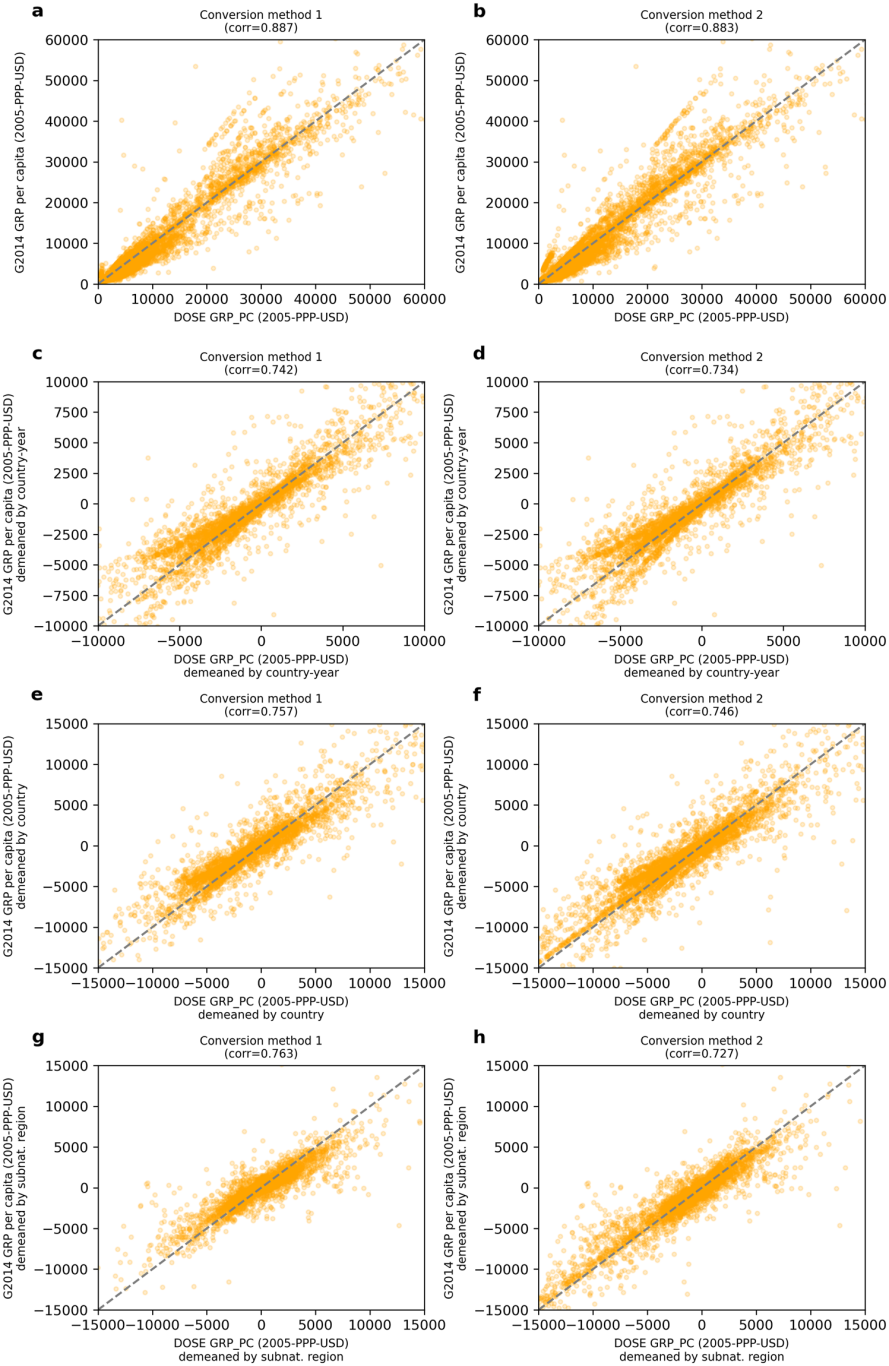
Comparison of DOSE to subnational data provided by Gennaioli *et al*. (G2014)^24^. For consistency with the methodology of G2014, values are PPP-adjusted and compared in constant 2005 International (PPP) dollars. Two different DOSE variables are used for the comparison: left panels show the case in which values were converted between currencies prior to the application of national deflators, whereas right panels show the case in which the order of these operations was reversed (note that neither method is directly equivalent to that applied by G2014^24^). Panels a and b compare the respective DOSE variable to that provided by G2014 across all regions and points in time. Panels c and d compare values from both sources having been demeaned by a country’s average GRP value in a given year to focus on consistency along the within-country dimension. Panels e and f compare values having been demeaned by a country’s average GRP in all years, to focus on both the within-country and temporal dimension. Panels g and h use values having been demeaned by a sub-national region’s average GRP in all years, to focus on the temporal dimension. Pearson coefficients of correlation are given in the respective panel title.

We identify approximately 5,600 observations of a total of 9,500 in G2014 which are comparable across both data sets, shown in Fig. 7 as scatterplots. The left four panels (a, c, e, and f) are based on the PPP-adjusted values estimated using the first method outlined in the section “Processing of Economic Data” (first converting to a common currency, then applying a US deflator, see eq. (1)). Conversely, the right hand panels (b, d, f and h) use the second conversion technique (first applying a national deflator, then converting to a common currency). In panels c and d, the comparison and correlation is made after both indicators have been demeaned by the national average GRP in each year, to emphasise consistencies and discrepancies between the two data sets in the within-country dimension. In panels e and f, this comparison is made after a demeaning by the national GRP value in all years to emphasise both the within-country and temporal dimension. In panels g and h, values are demeaned by the average GRP of each subnational region across all years to emphasise the temporal dimension only.

The comparison across countries (Fig. 7a,b, see Figure S3 in the Supplementary Information for a comparison on a logarithmic-scale) shows a high level of similarity and correlation (correlation coefficient, *r* > 0.88) for both conversion methods. High similarities (*r* > 0.7) are still obtained when comparing the data having been demeaned by country-year, country or sub-national region in order to focus on the within country and temporal dimensions (Fig. 7c–h). With regards to the different methods for currency conversion outlined in section “Economic Data Processing”, method 1 appears to provide generally greater consistency to the down-scaling method of G2014, particularly along the temporal dimension. When using non PPP-adjusted values to make this comparison, similarly high correlations are observed, with generally better correlations using the currency conversion order of method 2 (Figure S4 in the Supplementary Information). Overall, these comparisons validate the consistency of DOSE^17^ with previous estimates of sub-national output along multiple dimensions, strengthening confidence in our methodology and the additional 35,000 observations which DOSE^17^ provides in addition to theise previous data sets^24^.

### Limitations

There are a few limitations of DOSE^17^ that users should be aware of. Despite the increased spatial and temporal coverage of DOSE^17^ in comparison to most pre-existing datasets, data gaps in both dimensions remain. For example, we are still missing sub-national output data for a large number of African and Middle-Eastern countries (compare Fig. 2). This implies that regions in relatively wealthier countries are over-represented whereas poorer ones are under-represented. Temporally, our panel data also tends to be unbalanced with the majority of observations taking place over the last three decades of coverage (1990–2020) compared to the earlier decades (1960–1990). With respect to future developments, the use of satellite-derived data products could be a promising avenue for filling in spatial gaps.

Another limitation is that converting sub-national nominal GRP values in local currencies to real GRP data in USD is not straight-forward, partly due to the lack of auxiliary data at the sub-national level (such as GDP deflators which are generally unavailable at the global scale). Consequently, we provide a range of auxiliary data, allowing for different conversion procedures as described in detail in the Processing of Economic data (M2) section.

Finally, we acknowledge that the accuracy of DOSE^17^ is inherently limited by the accuracy and authenticity of national and regional administrations that report their economic output. The ‘reported’ nature of the values in DOSE^17^ should be emphasized in any future endeavors that exploit the data. It should be noted that some administrations may be incentivized to overestimate yearly GDP output, particularly in autocratic systems of governance^33^. Bearing this in mind, we believe there is great value in global databases of reported figures. Amongst other uses, they themselves form the basis of assessments of the integrity of reported figures and the integration of new observational methodologies such as nighttime light data.

## Usage Notes

Because of its.csv-format, the usage of DOSE is straight-forward. Researchers should be aware that when calculating sectoral shares of GRP, these should be assessed with respect to the sum of the three sectoral outputs. As the sectoral output data are generated by aggregating over specific categories of Gross Value Added by industry, GRP and the sum over the three sectors may not always equal one another. Regarding the different inflation and currency conversions, users can choose that economic variable which is most suitable for their research interest. The custom shapefile are provided in a.gpkg (geopackage).

## Supplementary information

Supplementary Table S1

Supplementary Information

## Data Availability

Code can be found in a public repository at: 10.5281/zenodo.7659599^34^. This includes code to replicate the figures shown here as well as code to merge spatial data from *GADM level 1* with the spatial data provided for eight exceptional countries as presented in Table 4 to produce a comprehensive shapefile with geometry for each region in DOSE.
