# Supplementary material for: DOSE – Global data set of reported sub-national economic output
Source: Sci Data. 2023 Jul 3;10:425. doi: 10.1038/s41597-023-02323-8 (PMC10318086; doi:10.1038/s41597-023-02323-8)
Supplement: Supplementary file 2 — Supplementary Information [file 41597_2023_2323_MOESM2_ESM.pdf]

Supplementary Information: DOSE – Global data set of reported sub-national economic output

Leonie Wenz[1,2,*], Robert Devon Carr[1,†], Noah Kögel[2,†], Maximilian Kotz[1,†], Matthias Kalkuhl[2,3]

1 Potsdam-Institute for Climate Impact Research (PIK)
2 Mercator Research Institute on Global Commons and Climate Change (MCC)
3 University of Potsdam, Faculty of Economics and Social Sciences
∗ correspondence to leonie.wenz@pik-potsdam.de
† these authors contributed equally to this work

This Supplementary Information document contains additional data visualizations and alternative plots related to the technical validation of DOSE. A full list of data sources by country can be found in Table S1 that is provided separately.

Table of Contents

1. Climate Data provided by DOSE 2
2. Technical Validation - Regional Coefficient of Variation 3
3. Technical Validation - Comparison with G2014 data 4

Climate Data provided by DOSE

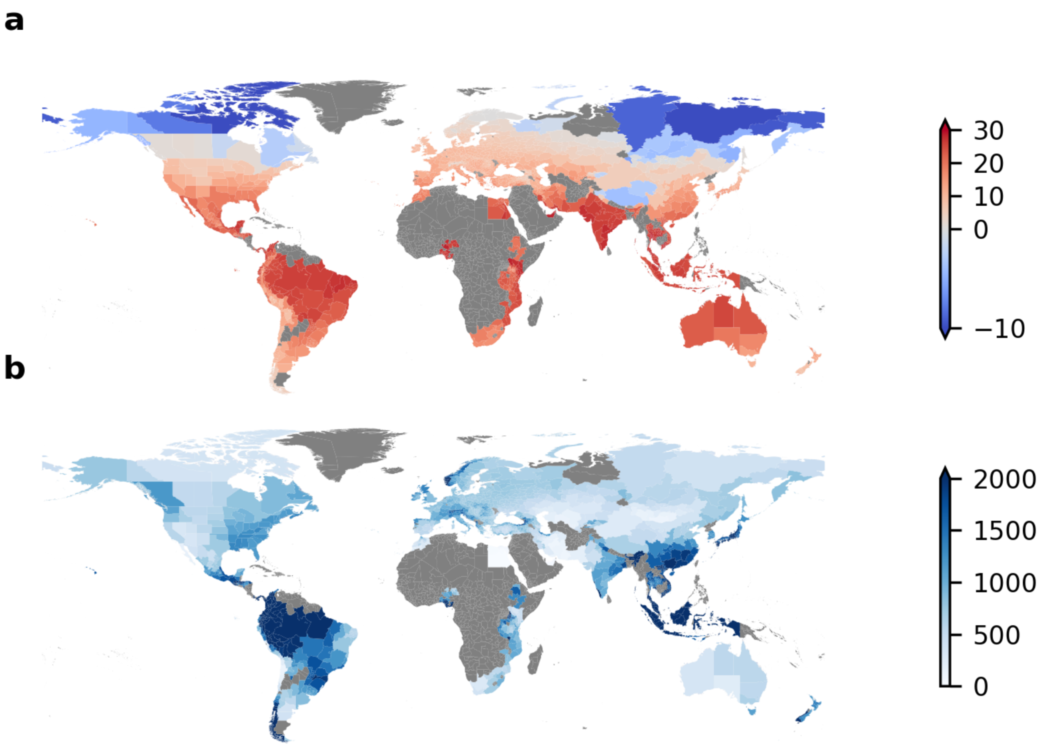

Figure S1: Climate data provided by DOSE. For each subnational region, the DOSE data set contains annual mean temperature and total annual precipitation data. They stem from the ERA5 reanalysis data set and have been aggregated from the grid cell (0.25°x0.25°) to the subnational level. Maps show the long-run annual mean temperature (panel a, in °C) and the long-run total annual precipitation (panel b, in mm).

Technical Validation - Regional Coefficient of Variation

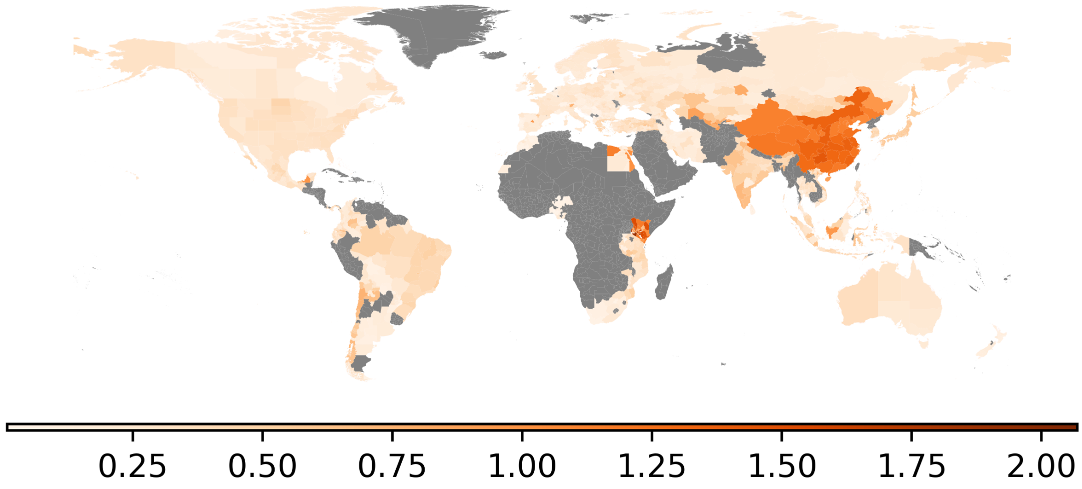

Figure S2: Regional coefficient of variation. The map shows each region's coefficient of variation, i.e. the ratio of the standard deviation to the mean, for DOSE's variable "grp_pc_lcu2015_usd" (per-capita GRP in in local 2015 prices, converted to US dollar using the 2015 exchange rate). Data appear to be consistent except for some outliers in e.g. China and Kenya which could be explained by either periods of large economic growth (China) or changes in the data source (Kenya), as explained in the Technical Validation section.

Technical Validation - Comparison with G2014 data

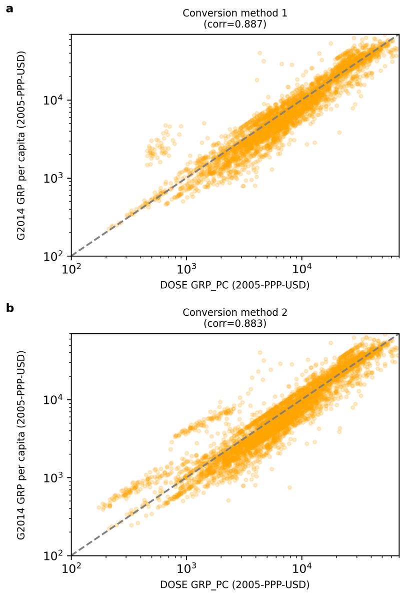

Figure S3: Comparison of DOSE to sub-national data as provided by Gennaioli et al 2014 (G2014). As Fig. 7a+b but with logarithmic scales to convey correspondence across the full distribution of values.

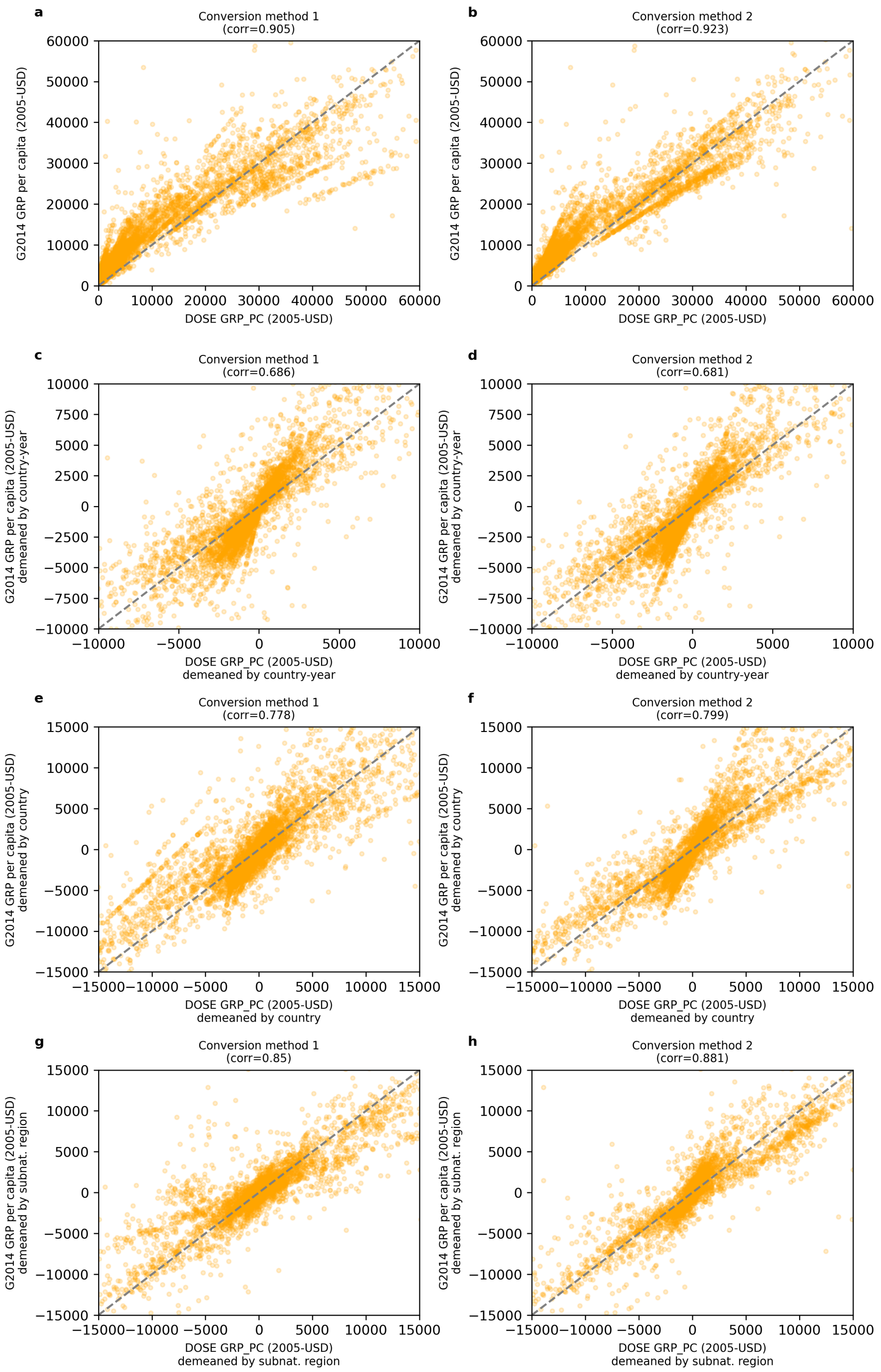

Figure S4: Comparison of DOSE to sub-national data as provided by Gennaioli et al 2014 (G2014) having used market exchange rates rather than PPP in the estimations of DOSE values. As Fig. 7 of the main manuscript but using market exchange rates rather than PPP.
